# Fetal double aortic arch: prenatal sonographic and postnatal computed tomography angiography features, associated abnormalities and clinical outcomes

**DOI:** 10.1186/s12884-020-03300-4

**Published:** 2020-10-12

**Authors:** Qiao Guo, Yifan Kong, Shi Zeng, Jiawei Zhou, Xiaofang Wang, Quanliang Shang, Jia Zhou, Hongxia Yuan, Ling Wang, Lili Tong, Aijiao Yi, Qichang Zhou

**Affiliations:** 1 Department of Ultrasonography, Second Xiangya Hospital of Central South University, 139 Renmin Road (M), 410011 Changsha, Hunan China; 2 Department of Obstetrics, Xiangya Hospital of Central South University, 87 Xiangya Road, 410008 Changsha, Hunan China; 3 Department of Radiology, Second Xiangya Hospital of Central South University, Changsha, Hunan China; 4 Department of Ultrasonography, the First Affiliated Hospital of South China University, Hengyang, Hunan China; 5 Department of Ultrasonography, Changsha Hospital for Maternal and Child Health Care, Changsha, Hunan China; 6 Department of Ultrasonography, Women and Children Healthcare Hospital of Zhuzhou, Zhuzhou, Hunan China; 7 Department of Ultrasonography, Maternal and Child Health Care Hospital of Changde, Changde, Hunan China; 8 Department of Ultrasonography, the First People’s Hospital of Yueyang, Yueyang, Hunan China

**Keywords:** Double aortic arch, vascular ring, fetal echocardiography, prenatal diagnosis, prenatal ultrasound

## Abstract

**Background:**

Fetal double aortic arch (DAA) malformation is a rare congenital heart disease with few reported cases in the literature. We aimed to investigate the characteristics of prenatal ultrasound and postnatal computed tomography angiography (CTA) of DAA and to describe the associated anomalies and clinical outcomes to improve prenatal diagnosis and assist in perinatal management.

**Methods:**

The obstetric ultrasound imaging databases of seven tertiary referral centers were reviewed retrospectively to identify fetuses with a prenatal diagnosis of DAA between January 2013 and December 2018. Ultrasonographic findings, associated anomalies, genetic abnormalities, postnatal CTA images, and long-term postnatal outcomes were evaluated.

**Results:**

A total of 36 cases out of 40 prenatally diagnosed DAA fetuses were confirmed by postnatal diagnosis (fetal autopsy, CTA, and surgery). In this cohort of 36 confirmed cases, 24 (67%) were isolated anomalies, while 12 (33%) were associated with intracardiac or extracardiac anomalies, and 2 (6%) had a 22q11.2 chromosome deletion. Among nine cases of pregnancy termination with a fetal autopsy, 7 had other abnormalities. Among the remaining 27 live births, 16 (59%) were asymptomatic and 11 (41%) received surgical treatment due to tracheal or esophageal compression symptoms, all with satisfactory outcomes. Prenatal echocardiography showed that DAA was mainly characterized by a bifurcation of the ascending aorta into the right and left aortic arch and the formation of a complete O-shaped vascular ring around the trachea on the three-vessel tracheal view. A variant in the aortic arch branching pattern was found for the first time. The airway obstruction, branching pattern, and atretic arch of DAA were clearly shown by postnatal CTA.

**Conclusions:**

Fetal DAA has unique features on prenatal echocardiography and postnatal CTA, and systematic prenatal examination and timely postnatal CTA evaluation are required. A certain proportion of intracardiac and extracardiac abnormalities are associated with DAA, but the probability of chromosome abnormalities is low, especially for isolated DAA.The clinical outcomes of isolated DAA are favorable, even if surgery is performed due to symptoms. Determining whether other malformations or chromosomal anomalies exist is crucial for prognosis evaluation and prenatal counseling.

## Background

A double aortic arch (DAA) is a rare type of congenital aortic arch anomaly, affecting approximately 0.005% ~ 0.007% of fetuses, while the prevalence of right aortic arch (RAA) is estimated to be 0.1% [1, 2]. If the growth of the bilateral fourth arch and dorsal aorta persists, a DAA forms [3]. DAA refers to the continuity of the left and right aortic arch. After arising from the ascending aorta, the two arches surround the trachea and esophagus to form a complete vascular ring and drains into the descending aorta. Generally, the left aortic arch gives rise to the left common carotid artery (LCCA) and the left subclavian artery (LSA), and the right aortic arch gives rise to the right common carotid artery (RCCA) and the right subclavian artery (RSA) [4]. Although the two arches are symmetrical, most cases will have one that is larger and extends higher toward the head. In rare cases, there is atresia of the arch, usually the left arch. The atretic segment is usually located at the distal end of the LSA but may also occur between the LCCA and LSA [5]. Generally, only one arterial duct is open, predominantly on the left, but a right or bilateral arterial duct has also been documented [6]. The descending aorta is usually located on the same side of the unobstructed arterial duct and opposite to the dominant aortic arch [7]. DAA with intracardiac malformations has been reported in 16.6% of cases, but extracardiac malformations have rarely been mentioned in previous studies. In a literature review including thirteen articles, only one DAA case with chromosome 22q11.2 deletion combined with extracardiac anomalies was reported [7]. Because of the vascular ring that encircles the trachea and esophagus, although some fetuses may be asymptomatic after birth, some m suffer from wheezing, dyspnea, dysphagia and other related compression symptoms [8].

Fetal DAA is generally diagnosed based on the characteristic complete vascular ring in the three-vessel tracheal view on ultrasound, and postnatal diagnosis can be confirmed by computed tomography angiography (CTA) [1, 9]. At present, reports in the literature on DAA are mostly about children or adults, and the reports addressing fetuses all have small sample sizes, with the largest study comprising only 9 cases [10]. Due to the few studies with large sample sizes and the lack of detailed systematic examination approaches, an accurate and comprehensive prenatal diagnosis of DAA remains difficult, and misdiagnoses or missed diagnoses can easily occur. A retrospective analysis of fetal DAA was performed in our study to investigate the prenatal ultrasound and postnatal CTA characteristics of DAA and to describe the associated anomalies and clinical outcomes to improve prenatal diagnosis and assist in perinatal management.

## Methods

All cases of prenatally suspected diagnosis of DAA in the fetal ultrasound examination databases of seven tertiary referral centers from January 2013 to December 2018, were retrospectively retrieved. Other postnatally diagnosed DAA cases without a prenatal ultrasound diagnosis were excluded in the study. Prenatal and postnatal medical records, including echocardiographic and CT scan images, videos and reports, fetal autopsy findings, neonatal records of the newborns, and operation records, were reviewed. Baseline demographics (including maternal age, gestational age at diagnosis, follow-up period, et al.), prenatal sonographic findings, associated cardiac or extracardiac abnormalities, genetic testing results, and pregnancy outcomes were recorded. Data from postnatal CTA imaging, autopsy, and corrective surgery for postnatal confirmation of the diagnosis were retrieved and analyzed. For fetuses born alive, symptoms related to compression of the airways/esophagus and the outcomes of surgery for vascular ring were evaluated. In addition, the cases lost to follow-up and misdiagnosed cases were also documented. This study was approved by the hospital ethics committee, and informed consent was obtained from the pregnant women.

The high-quality GE Voluson E10, GE Voluson E8 Expert, and Toshiba Aplio500 Color Doppler ultrasound diagnostic instruments were used, and the probes equipped were RM6C, RAB4-8-d, C1-5-D, and PVT-375BT. Imaging was performed with fetal OB examination mode with the thermal index and medical index each set at < 1.0 and the ALARA principle was followed. Each pregnant woman was positioned in the supine or lateral position, underwent a routine obstetric ultrasound examination and then underwent a fetal cardiac assessment in which a complete fetal echocardiography by a fetal echocardiography expert was performed based on the ISUOG Practice Guidelines (updated) for sonographic screening examination of the fetal heart [11]. Volume sonography (spatiotemporal image correlation (STIC)) could also be incorporated into a more detailed anatomical and functional assessment of the fetal heart if necessary, as previously described [12]. Under normal circumstances, the three-vessel tracheal view shows that the aortic arch and ductus arteriosus together form a V shape, converging into the descending aorta and that the trachea is located posteriorly and to the right, with no vascular constriction in front of the trachea. The diagnosis of DAA was made on the presence of two aortic arches, one on each side of the trachea, and forms a complete vascular ring, joining posteriorly to the descending aorta, with the common carotid and subclavian arteries arising separately and symmetrically, one from each arch. The laterality of the arterial duct in relation to the trachea was also ascertained. After the determination of DAA malformation, the inner diameter of the aortic arch was measured. According to the Backer’s classification standard, DAA was divided into the right arch dominant type, left arch dominant type and double arch balanced type [13]. On this basis, we set the ratios of the inner diameter of the right arch to that of the left arch as follows: between 0.9 and 1.1 for a double arch balanced type, greater than 1.1 for a right arch dominant type and less than 0.9 for a left arch dominant type.

For fetuses with ultrasonographic findings of DAA, careful examination was carried out to determine whether there was any other intracardiac or extracardiac abnormality. Isolated DAA refers to the absence of intracardiac and extracardiac abnormalities. All parents received detailed counseling regarding the diagnosis and therapeutic options after the ultrasound scan. Karyotyping and genetic testing with chromosomal microarray analysis (CMA) by amniotic fluid or umbilical cord blood puncture was suggested. With the consent of the parents of the fetuses, autopsy and pathological examinations were performed for those who chose to terminate their pregnancies, and follow-up was conducted after delivery for those who continued their pregnancies.

For every pediatric patient, multidetector-row computed tomography (MDCT) angiography examinations were performed with a 320-detector volume CT system (Aquilion ONE, Toshiba, Japan) or a 256-row MDCT (GE Revolution, USA). Patients fasted for 46 h and were then anesthetized (chloral hydrate: 0.5 mg/kg). Imaging data were acquired after an intravenous injection of 1.5-2 ml/kg nonionic iodinated contrast agent (iopromide; Ultravist; Schering AG, Berlin, Germany) at a rate of 2-2.5 ml/s. For three-dimensional image reconstruction, the raw MDCT data were processed on a separate workstation (VITREA or Advanced Workstation 4.7, GE Revolution) with multiplanar reformatting (MPR), maximum intensity projection (MIP), minimum intensity projection (MinIP) and volume rendering (VR).

Statistical analysis was performed with Microsoft Excel for Windows 7 (version 14.0.4760.1000). Continuous variables were presented with the mean ± standard deviation (SD), and categorical variables were presented with proportions (percentage).

## Results

### Baseline characteristics of prenatally diagnosed DAA cases

**Fig. 1:**
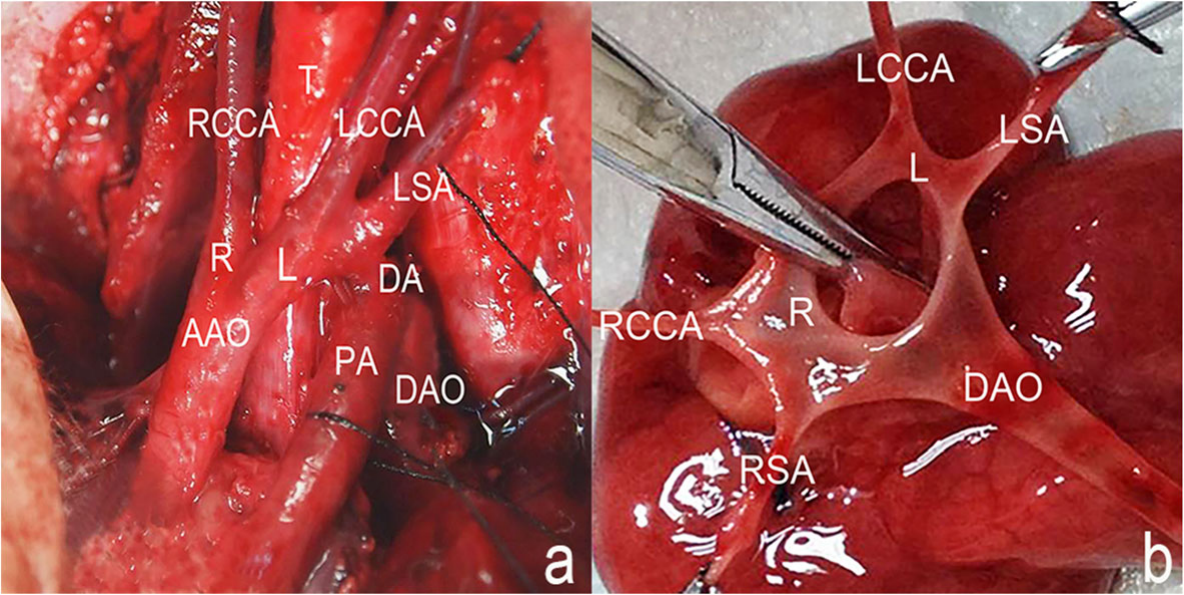
Fetal anatomical pathology after termination of pregnancy. **a** The right and left aortic arches both arise from the ascending aorta. The left aortic arch gives rise to the LCCA and LSA, and the right aortic arch gives rise to the RCCA and RSA. The left ductus arteriosus connects to descending aorta. **b** After the trachea is cut off, the left and right arches form a complete vascular ring, which is connected to the descending aorta. (AAO: ascending aorta; R: right aortic arch; L: left aortic arch; RCCA: right common carotid artery; RSA: right subclavian artery; LCCA: left common carotid artery; LSA: left subclavian artery; PA: pulmonary artery DA: ductus arteriosus; DAO: descending aorta; T: trachea)

A total of 358,815 fetuses with complete prenatal ultrasound data from seven centers during six years were analyzed, among which 40 cases were diagnosed with DAA prenatally and 36 cases had postnatal confirmation (9 cases by autopsy (Fig. 1) and 27 cases by CTA; 11 cases were further confirmed by cardiac surgery), with an incidence of 0.01% (36/358,815). There were two misdiagnosed cases, a right aortic arch with mirror-imaging branching and a left posterior ductus arteriosus connecting the descending aorta (MRAA- LPDA-DAO) and a right aortic arch with a left posterior ductus arteriosus and an aberrant left subclavian artery (RAA-LPDA-ALSA), respectively. In addition, one case of pregnancy termination lacked an autopsy, and one case was lost to follow-up (Table 1). The mean maternal age was 29 (range, 23–35) years, and the mean gestational age (GA) was 27 (range, 23–31) weeks.

**Table 1:** Diagnostic accuracy of prenatal ultrasound in the fetal DAA cohort (*n* = 40)

	N (%)
**Confirmed diagnosis**	36 (90%)
▲Autopsy	9/36 (25%)
▲Live births	27/36 (75%)
▲▲CTA	27/27 (100%)
▲▲CTA, and surgery	11/27 (41%)
**Misdiagnosis**	2 (5%)
▲MRAA- LPDA-DAO	1 (3%)
▲RAA-LPDA-ALSA	1 (3%)
**Lost to follow-up**	1 (3%)
**Lack of autopsy**	1 (3%)

*DAA* double aortic arch, *CTA* computed tomography angiography, *MRAA- LPDA-DAO* right aortic arch with mirror-image branching and a left posterior ductus arteriosus connecting the descending aorta. *RAA-LPDA-ALSA* right aortic arch with a left posterior ductus arteriosus and an aberrant left subclavian artery

### Associated abnormalities and pregnancy outcomes of DAA fetuses

According to the Backer’s classification and specific standards mentioned above, among the 36 fetuses, 25 (69%) were right arch dominant, including 2 with an atretic left arch; 5 (14%) were left arch dominant, and 6 (17%) were double arch balanced (Table 2). All cases had left-sided ductus arteriosus. Of the 36 DAA cases, 24 (67%) were isolated abnormalities, 11 (31%) were associated with intracardiac malformations, and 5 (14%) were associated with extracardiac malformations, among which 4 (11%) had intracardiac and extracardiac malformations simultaneously (Tables 2 and 3). Karyotyping and genetic testing with chromosomal microarray analysis (CMA) by amniotic fluid or umbilical cord blood puncture were performed in 33 cases, and two cases of chromosome 22q11 deletion were identified, both of which were associated with simultaneous intracardiac and extracardiac abnormalities. Finally, 9 pregnancies were terminated, and 27 fetuses were delivered alive. Among the 9 fetuses with TOP, 7 had intracardiac or extracardiac malformations (of which 2 were associated with 22q11 deletion), and 2 were isolated DAA. Of the 27 live births, 16 (59%) were asymptomatic during the study period, and 11 (41%) suffered from varying degrees of stridor, dyspnea, recurrent upper respiratory tract infections and dysphagia for surgical treatment. All of them survived with good prognoses. Among the 11 babies who underwent surgery, 7 were isolated DAA, and 4 were associated with intracardiac or extracardiac abnormities, namely, a ventricular septal defect in 1 case, a permanent left superior vena cava in 2 cases, and a diaphragmatic hernia in 1 case. The mean follow-up duration was 38.0 ± 17.0 months and the mean age at symptom occurrence was 4.0 ± 2.8 months (Table 2).

**Table 2:** The features and outcomes of the fetal DAA group with a postnatally confirmed diagnosis (*n* = 36)

Characteristics	Mean ± SD or N(%)
Maternal age, y	29.0 ± 6.0
Gestational age at diagnosis, wks	27.0 ± 4.1
Follow-up period, months	38.0 ± 17.0
Age of symptoms, months	4.0 ± 2.8
Backer’s Classification	/
▲Right arch dominant	25 (69%)
▲▲Atretic left arch	2 (6%)
▲Left arch dominant	5 (14%)
▲Double arch balanced	6 (17%)
Isolated DAA	24 (67%)
Intracardiac anomalies	11(31%)
Extracardiac anomalies	5 (14%)
Chromosomal abnormalities	2/33 (6%)
TOP	9(25%)
Live births	27 (75%)
**▲**Asymptomatic	16/27 (59%)
**▲**Symptomatic for surgery	11/27 (41%)

*DAA* double aortic arch, *TOP* termination of pregnancy

**Table 3:** Associated anomalies in the fetal DAA group with a postnatally confirmed diagnosis (*n* = 36)

	N (%)
**Cardiac**	11 (31%)
▲Ventricular septal defect	5 (14%)
▲Double outlet right ventricle	4 (11%)
▲Permanent left superior vena cava	4 (11%)
▲Left ventricular dysplasia	1 (3%)
▲Partial atrioventricular septal defect	1 (3%)
▲Mirror image dextrocardia	1 (3%)
▲Dextrocardia	1 (3%)
**Extracardiac**	5 (14%)
▲Gastrointestinal tract	2 (6%)
▲Thorax	2 (6%)
▲Central nervous system	1 (3%)
▲Facial	1 (3%)
▲Spine	1 (3%)

*DAA *double aortic arch

### Main echocardiography characteristics of DAA fetuses

**Fig. 2:**
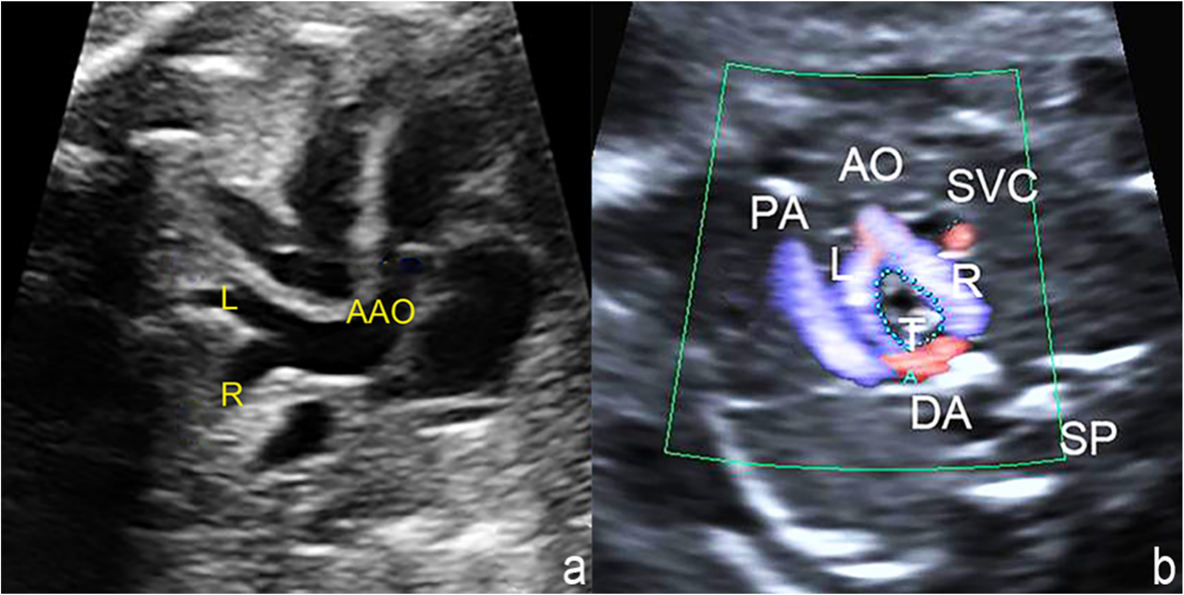
Bifurcation of the ascending aorta and a complete vascular ring of the DAA on fetal echocardiography. **a** Left ventricular outflow tract view: The distal ascending aorta bifurcation is confirmed as the right arch and left arch. **b** Three-vessel tracheal view: A bifurcation of the ascending aorta into the right aortic arch and left aortic arch to form a complete O-shaped ring encircling the trachea, together with a number 9 configuration connecting with the left-sided ductus arteriosus. Both aortic arches demonstrate antegrade blood flow (AO or AAO: ascending aorta; R: right aortic arch; L: left aortic arch; PA: pulmonary artery; DA: ductus arteriosus; T: trachea)

**Fig. 3:**
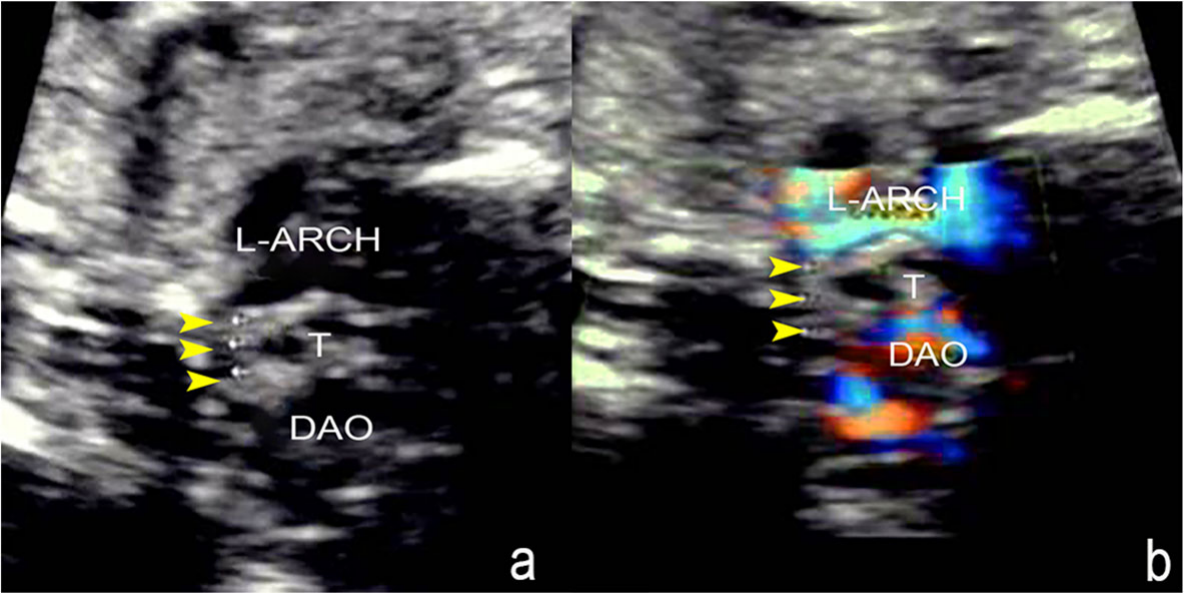
DAA with distal left aortic arch atresia on fetal echocardiography. **a** Nonstandard three-vessel tracheal view of gray-scale imaging: The vascular echo at the distal end of the left aortic arch is interrupted and replaced by a fibrous cord (arrowhead). **b**. Nonstandard three-vessel tracheal view of color Doppler imaging shows the interruption of color flow at the distal end of the left aortic arch(arrowhead) and an incomplete vascular ring(L-ARCH: left aortic arch; DAO: descending aorta; T: trachea)

**Fig. 4:**
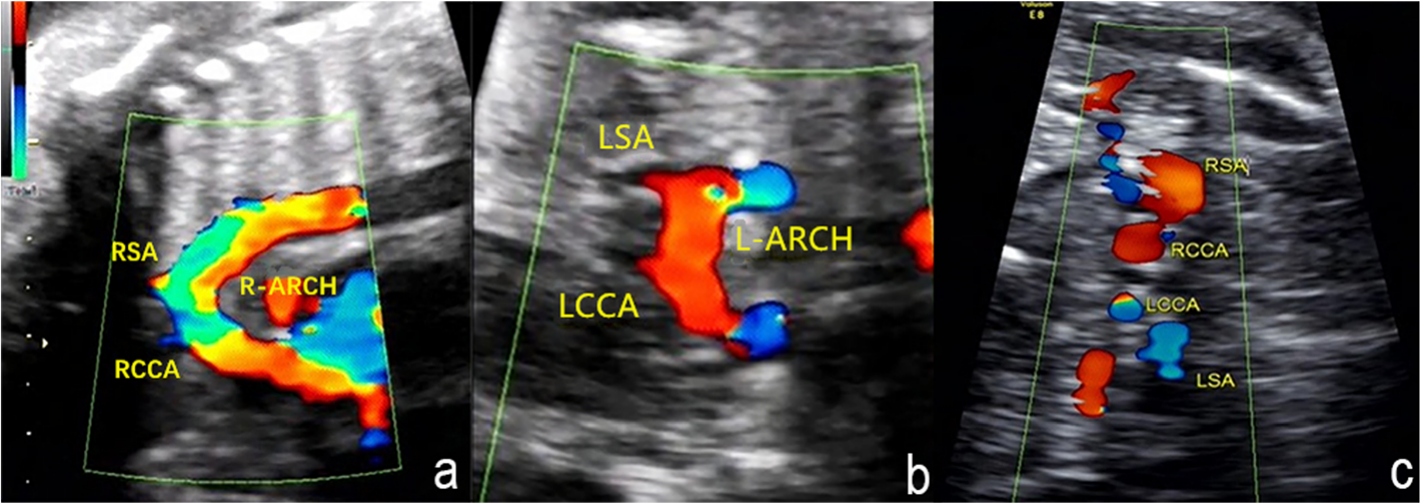
Branching pattern of DAA on fetal echocardiography. **a** Sagittal view of the right aortic arch: The dominant right arch gives rise to the RCCA and RSA. **b** Sagittal view of the left aortic arch: The small left aortic arch gives rise to the LCCA and LSA. **c** The coronal view of the aortic arch shows the symmetric appearance of the common carotid artery and subclavian artery originating from the ipsilateral aortic arch simultaneously(R-ARCH: right aortic arch; L-ARCH: left aortic arch; RCCA: right common carotid artery; RSA: right subclavian artery; LCCA: left common carotid artery; LSA: left subclavian artery)

**Fig. 5:**
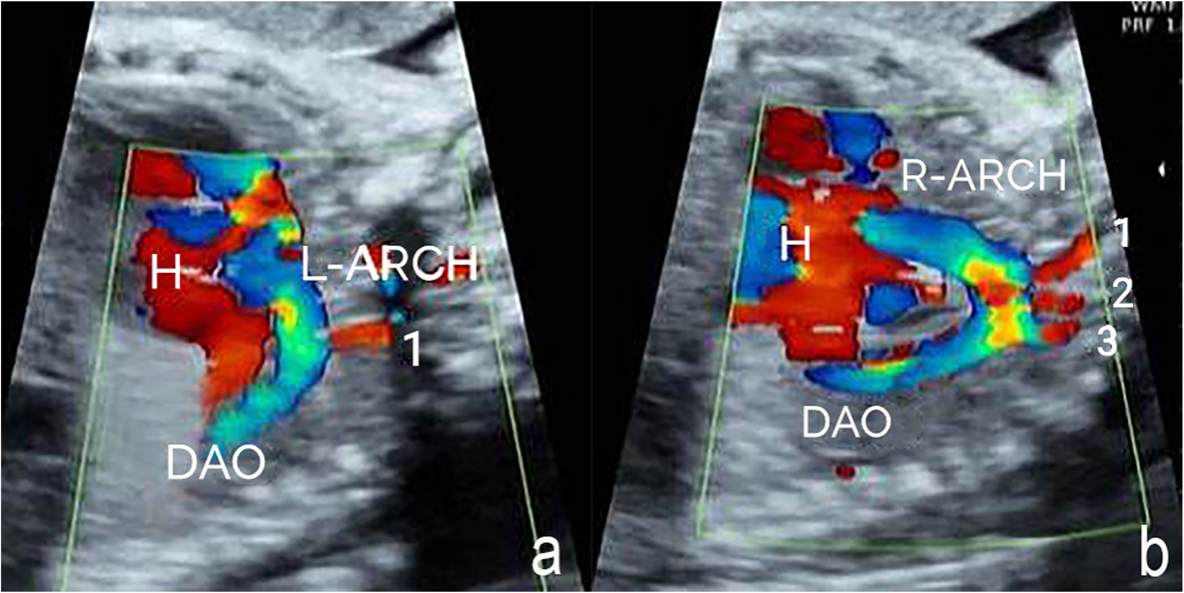
Branching pattern variant of DAA on fetal echocardiography. **a** Sagittal view of the left aortic arch: One branch can be viewed from the left aortic arch (H: heart, 1: left common carotid artery). Sagittal view of the right aortic arch: Three branches can be viewed from the right aortic arch (H: heart, 1: right common carotid artery, 2: right subclavian artery, 3: left subclavian artery, L-ARCH: left aortic arch; R-ARCH: right aortic arch; DAO: descending aorta)

**Fig. 6:**
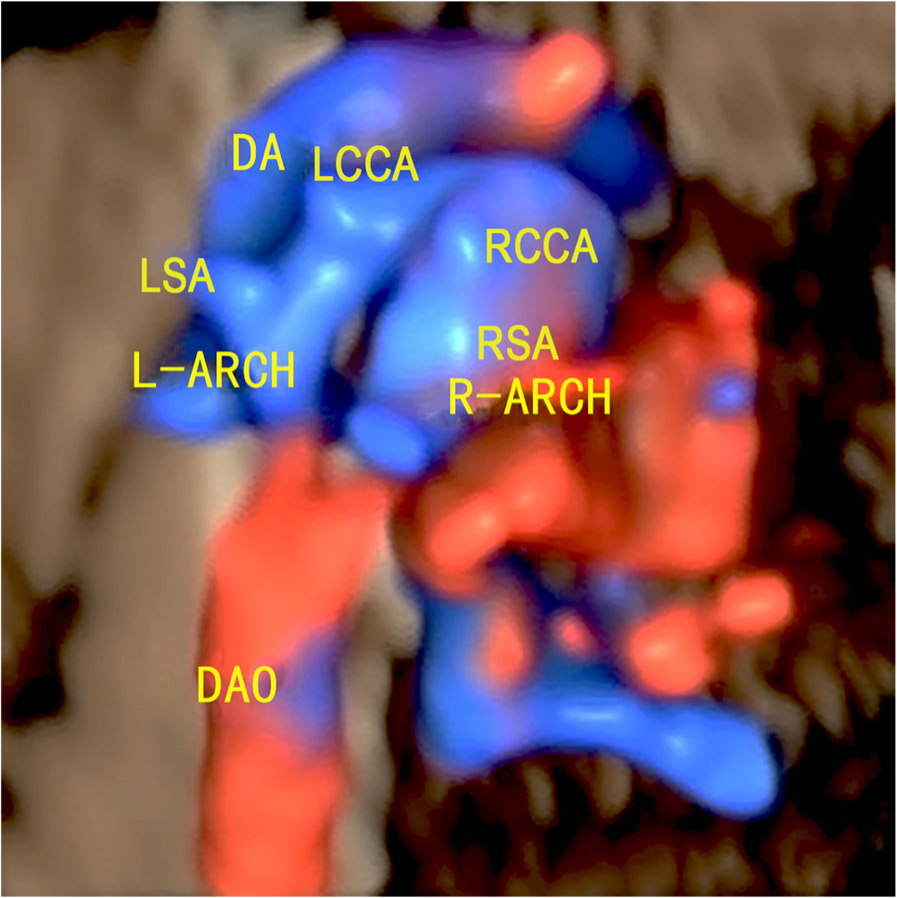
Three-dimensional color-rendered image with spatiotemporal image correlation of DAA: The left aortic arch and the dominant right aortic arch develop a complete vascular loop, join the left ductus arteriosus, and converge into the descending aorta together. Symmetrical initial parts of the two branches can probably be observed on each arch. (R-ARCH: right aortic arch; L-ARCH: left aortic arch; RCCA: right common carotid artery; RSA: right subclavian artery; LCCA: left common carotid artery; LSA: left subclavian artery; DA: ductus arteriosus; DAO: descending aorta)

The main echocardiography characteristics of DAA are as follows: (1) A bifurcation of the ascending aorta into the right and left arch is confirmed on the left ventricular outflow tract view and the three-vessel tracheal view (Fig. 2a and b); (2) For a right-sided arch, an aortic arch is visible on the right side of the trachea on the three-vessel tracheal view (Fig. 2b); (3) The three-vessel tracheal view can show that the left and right arches form a complete O-shaped vascular ring around the trachea and that the ductus arteriosus is connected to it to develop a 9-shaped configuration (Fig. 2b). However, in the two cases with an atretic arch, the color flow is interrupted and the O-shaped vascular ring is not completed, and a fibrous cord could be seen (Fig. 3); (4) The three-vessel tracheal view or sagittal view may show that both the left and right arches converge into the descending aorta (Figs. 2b and 4a and b); (5) The sagittal view shows two aortic branches (the LCCA and LSA) of the left arch and two symmetrical branches (the RCCA and the RSA) of the right arch (Fig. 4a and b); (6) The coronal view of the aortic arch shows the symmetric appearance of the common carotid artery and subclavian artery originating from the ipsilateral aortic arch simultaneously (Fig. 4c). However, a variant in the aortic arch branching pattern was found in one case in the examination, namely, only one branch of the left aortic arch and three branches of the right aortic arch (Fig. 5); and (7) Three-dimensional color-rendered images with spatiotemporal image correlation may help display the spatial structure of the complete vascular ring and branches (Fig. 6).

### Postnatal CTA results and main features

**Fig. 7:**
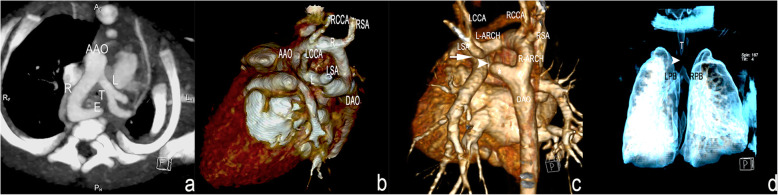
Postnatally confirmed diagnosis of DAA by multidetector-row CTA with volume-rendering processing. **a** Transaxial view: The ascending aorta divides into two arches surrounding the trachea and esophagus. **b** Left view of volume-rendered image: The spatial structure mimics that in Fig. 6, but the branches (the LCCA and the LSA originating from the left arch, the RCCA and the RSA originating from the right arch) and the arches surrounding of the trachea and esophagus appear clearer. **c** The proximity of the posteroinferiorly distorted left subclavian artery (arrow) and descending aortic diverticulum (*) suggests a possible fibrous connection (arrowhead) between the two structures and demonstrates a double aortic arch with an atretic left aortic arch distal to the origin of the left subclavian artery. d. A trachea compressed by the vascular ring is shown (arrowhead) (AAO: ascending aorta; R or R-ARCH: right aortic arch; L or L-ARCH: left aortic arch; RCCA: right common carotid artery; RSA: right subclavian artery; LCCA: left common carotid artery; LSA: left subclavian artery; DAO: descending aorta; T: trachea; LPB: left principal bronchus, RPB: right principal bronchus)

A total of 29 infants underwent CTA between 1 week and 1 year after birth. Twenty-seven cases were confirmed to be DAA, including 2 cases with atresia of the left arch. Two cases were found to be an MRAA-LPDA-DAO and an RAA-LPDA-ALSA. Of the 27 cases, 11 with symptoms of tracheal or esophageal compression showed a tracheal or esophageal obstruction on CTA. The main features of DAA on CTA are as follows: (1) On the transaxial view with MIP, the ascending aorta divides into two arches surrounding the trachea and esophagus, which mimic the prenatal three-vessel tracheal view on ultrasound (Fig. 7a); (2) VR image processing for great vessels clearly shows the double arches forming a complete vascular ring and both connecting with the descending aorta and two branches arising from each arch (Fig. 7b), and a proximal posteroinferiorly distorted LSA and descending aortic diverticulum indicates a DAA with an atretic left aortic arch in two cases (Fig. 7c); and (3) MPR or MinIP or VR image processing for the airway can accurately demonstrate the site and severity of the tracheal obstruction in 11 cases (Fig. 7d).

## Discussion

To the best of our knowledge, this is the largest multicenter retrospective cohort study investigating the natural history of DAA, prenatal ultrasound and postnatal CTA characteristics, associated abnormalities, and clinical outcomes. These cases were all thoroughly followed up and had postnatal confirmation. From an anatomical point of view, we report a new branching variant of DAA for the first time. In contrast to the usual pattern wherein each aortic arch gives rise to two branches, in this case, only one branch originated from the left aortic arch, namely, the LCCA, but three branches originated from the right aortic arch, namely, from proximal to distal, the RCCA, RSA, and LSA. However, additional similar cases are needed to confirm this discovery.

DAA is a mostly isolated abnormality but can also be associated with other abnormalities. We found that 31% of cases had DAA with intracardiac malformations, in contrast to 16.6% of cases in another report [7], with ventricular septal defect, double outlet right ventricle, and persistent left superior vena cava being the most common. Extracardiac malformations were rarely mentioned previously [7], but 5 cases (14%) were found in this study, highlighting that a careful and thorough fetal examination outside of the cardiovascular system is also needed. The prevalence of chromosome 22q11.2 microdeletions (DiGeorge syndrome) in fetuses with RAA has been reported to be between 6.1% and 10% [14–16]. Compared to RAA, DAA was associated with a smaller proportion of 22q11 deletion (6%), which is especially rare in isolated DAA, and the two cases of 22q11.2 chromosome deletion in this study were both accompanied by intracardiac malformations and extracardiac malformations (including thymus dysplasia). Ultrasound assessment of dysplasia or the absence of a fetal thymus is useful for predicting 22q11.2 microdeletion [17]. Therefore, for fetuses diagnosed with DAA by ultrasound, thymus size assessment should be performed routinely. In this study, 9 women had terminations of pregnancy, among which 7 were associated with intracardiac or extracardiac malformations or 22q11.2 microdeletion, indicating that whether DAA is associated with other malformations or chromosomal abnormalities has an important impact on pregnancy outcomes and maternal decision-making.

Based on the abovementioned sonographic characteristics of DAA, most DAAs can be diagnosed by prenatal echocardiography. The three-vessel tracheal view is the most characteristic view for DAA diagnosis, but it alone is not enough, especially when an arch shows atresia, in which a vascular ring is not always typical as a result of an interruption in the blood flow of the atretic segment and then differential diagnosis with a right aortic arch is challenging. At this time, multiple views should be performed to better observe the origin, course, and branches of the two arches. The following steps and key points for systematic examination are recommended. (1) Whether the right aortic arch is present on the three-vessel tracheal view should be confirmed. (2) A search for bifurcation of the ascending aorta in the left ventricular outflow tract view is needed; it is necessary to trace the ascending aorta far enough into the arch as the bifurcation is usually not at the origin of the ascending aorta. (3) The three-vessel tracheal view and sagittal view of the aortic arch should be used to confirm that the left and right arches both arise from the ascending aorta and are connected to the descending aorta. (4) The three-vessel trachea view should be used to find the left and right arches surrounding the trachea and esophagus to form a complete O-shaped vascular ring; because the two arches may not be at the same level, the probe will need to be tilted slightly. (5) To determine the type of DAA, the inner diameter of the left and right arches need to be accurately measured. (6) The branching patterns of the left and right aortic arches on the sagittal views and coronal views of the aortic arch need to be confirmed. (7) Careful examination of the heart and other systems, including the thymus, should be performed to determine the existence of associated intracardiac or extracardiac malformations. (8) Chromosome and gene tests should be performed when necessary. Besides, adjusting the parameters of the color Doppler mode is necessary to reduce the scale of blood flow velocity to display a small nondominant arch fully. It is difficult to identify the fibrous cord formed by partial atresia of the nondominant arch by ultrasound examination, so the vascular ring needs to be diagnosed indirectly through the formation of a blind end or diverticulum of the arch. When the left arch is small and difficult to display, the only visible right arch should not be mistaken for the right pulmonary artery, which would also be going in the right direction.

Regarding cases before birth, very few reports have suggested that tracheal compression by the complete vascular ring of the DAA could lead to CHAOS (congenital high airway obstruction syndrome), which can cause intrauterine fetal respiratory distress or stillbirth [10, 18]. However, the sonographic characteristics for predicting perinatal complications of DAA, especially for tracheal compression requiring airway ex utero intrapartum therapy (EXIT), are not clear. In our study, none of the fetuses showed significant airway obstruction before delivery, consistent with most reports.

After birth, some DAA patients have compression due to the vascular ring surrounding the trachea and esophagus, resulting in wheezing, dyspnea, dysphagia and other symptoms, most of which occur within the first year. Currently, it is believed that surgical treatment is necessary for patients with respiratory or digestive symptoms [8, 19]. The proportion of children with symptomatic DAA is reported to be approximately 72.4% [7]. However, during the follow-up after birth in this study, 16 cases (59%) were asymptomatic, and 11 symptomatic cases (41%) underwent surgical treatment. From our study, DAA seemed to have a more aggressive clinical presentation (41% vs. 5.6%-25.2%) and usually required surgical intervention (41% vs. 5.6%-17.1%), as opposed to other forms of RAA [2, 14, 20].

Echocardiography is considered to be the first-line postnatal imaging method for DAA. However, rings with special anatomical features, such as a fibrous cord and tracheal compression, cannot be recognized by ultrasound, and pulmonary air easily interferes with image quality. Therefore, MRI or CT is considered to be the gold standard for identifying such variations. However, MRA may require prolonged sedation of pediatric patients [21]. Moreover, image reconstruction, density, and time resolution are worse with MRA than with CT. Compared to MRA, MDCT is a superior imaging modality as it requires less time for a child to calm down and provides more detailed information, including vascular structures and spatial relationships with adjacent organs, especially the airways and esophagus [22]. MDCT combined with various postprocessing options, such as VR, MIP, MinIP, and MR, can display clear details of the compressed trachea, esophagus, and vascular ring, even in cases in which the ring comprises the atretic aortic arch and arterial ligament. Generally, evidence of inferior and posterior convexity of the initial course of the LSA and a descending aortic diverticulum suggests the presence of an imperforate vessel or fibrous cord connecting the structures of the atretic aortic arch [23].

The two cases of misdiagnosis in this study should be noted. Except for the familiar RAA-LPDA-ALSA forming a U-shaped vascular ring, DAA, especially the dominant right arch type, should also be identified with MRAA-LPDA-DAO. When the left arch is small and tortuously curved, it is easily mistaken for the left innominate artery, and the O-shaped vascular ring is not obvious and typical. Repeated multiple cross-sectional examinations show that MRAA-LPDA-DAO fails to demonstrate the connection between the left branch of the aortic arch (the left innominate artery) and the ductus arteriosus or descending aorta. However, in DAA, the left arch is usually connected with the left ductus arteriosus and descending aorta.

Regarding prenatal counseling, we offer some suggestions. First, although DAA was mostly isolated, a certain proportion of intracardiac and extracardiac abnormalities may be associated with it. Therefore, systematic and comprehensive anatomical ultrasonic screening for DAA fetuses is essential. DAA fetuses with intracardiac or extracardiac malformations should be evaluated in detail to assess the severity of the combined malformations and inform the mothers of the prognosis. Secondly, invasive prenatal genetic diagnosis is recommended for DAA fetuses with other intracardiac or extracardiac abnormalities. However, for isolated DAA, genetic testing is not all required and can be discussed with patients. Termination of pregnancy is recommended for fetuses with chromosomal abnormalities (mainly 22q11.2 microdeletion), which often lead to a severe syndrome. Finally, fetal retention is recommended for DAA without severe associated abnormalities and chromosomal abnormalities as the clinical outcomes are favorable, even if surgery is performed due to compression symptoms. However, timely CTA examination for a definite diagnosis and accurate postnatal evaluation is needed.

The main strengths of the study lie first in its large sample size, reliable postnatal confirmation diagnosis and an adequate follow-up duration. In addition, a detailed prenatal ultrasound and postnatal CTA assessment were performed. For the first time, we systematically propose the steps and key points for prenatal ultrasound diagnosis of DAA to improve prenatal diagnosis. And through analyzing the features and advantages of postnatal CTA images, we recommend timely application of CTA examination for accurate postnatal evaluation. Finally, our study provides useful information for prenatal counselling of DAA fetuses.

However, several limitations of this research are worthy of note. First, the incidence of DAA in our study (0.01%), was higher than that in the unselected population according to the literature (0.005%-0.007%) [1, 2]. This discrepancy may be because the research centers in this study were provincial or municipal tertiary referral centers, where many women with high-risk pregnancies or who were suspected of a fetal heart anomaly were referred and selectively examined. Moreover, since we included only fetuses with prenatal diagnoses of DAA, false-negative diagnoses could not be derived and analyzed. Finally, we tried to find useful ultrasonographic manifestations that can predict respiratory symptoms after birth but failed, so further prospective studies are needed.

## Conclusions

In summary, fetal DAA has unique features on prenatal echocardiography and postnatal CTA images. A detailed and systematic prenatal ultrasound examination may effectively improve the accuracy of the prenatal diagnosis and CTA may be the optimal imaging method to evaluate DAA postpartum. DAA is a mostly isolated abnormality but a certain proportion of intracardiac and extracardiac abnormalities are associated with it. The probability of chromosome abnormalities is very low, especially for isolated DAA. Most cases are asymptomatic after birth, and the clinical outcomes are favorable, even if surgery is performed due to tracheal or esophageal compression symptoms. Determining whether other malformations or chromosomal anomalies exist is crucial for prognostic evaluations and prenatal counseling.

## Data Availability

All data generated or analysed during this study are included in this published article. The datasets used and/or analysed during the current study are available from the corresponding author on reasonable request.

## Abbreviations

DAA: double aortic arch
CTA: computed tomography angiography
RAA: right aortic arch
STIC: spatiotemporal image correlation
CMA: chromosomal microarray analysis
MDCT: multidetector-row computed tomography
AAO: ascending aorta
RCCA: right common carotid artery
RSA: right subclavian artery
LCCA: left common carotid artery
LSA: left subclavian artery
DA: ductus arteriosus
DAO: descending aorta
PA: pulmonary artery
MRAA- LPDA-DAO: right aortic arch with mirror-image branching and a left posterior ductus arteriosus connecting the descending aorta
RAA-LPDA-ALSA: right aortic arch with a left posterior ductus arteriosus and an aberrant left subclavian artery
MPR: multiplanar reformatting
MIP: maximum intensity projection
MinIP: minimum intensity projection
VR: volume rendering
SD: standard deviation
CHAOS: congenital high airway obstruction syndrome
EXIT: ex utero intrapartum therapy
